# Association of the thyroid hormone responsive spot 14 alpha gene with growth-related traits in Korean native chicken

**DOI:** 10.5713/ajas.19.0541

**Published:** 2020-02-25

**Authors:** Muhammad Cahyadi, Hee-Bok Park, Dong Won Seo, Shil Jin, Nuri Choi, Kang Nyeong Heo, Bo Seok Kang, Cheorun Jo, Jun Heon Lee

**Affiliations:** 1 Department of Animal Science, Faculty of Agriculture, Universitas Sebelas Maret, Surakarta 57126, Indonesia; 2 Department of Animal Resources Science, Kongju National University, Yesan 32439, Korea; 3 Department of Animal and Dairy Science, College of Agriculture and Life Sciences, Chungnam National University, Daejeon 34134, Korea; 4 Hanwoo Research Institute, National Institute of Animal Science, RDA, Pyeongchang 25340, Korea; 5 Division of Biotechnology, College of Environmental and Bioresource Sciences, Chonbuk National University, Iksan 54596, Korea; 6 Poultry Research Institute, National Institute of Animal Science, RDA, Pyeongchang 25340, Korea; 7 Department of Agricultural Biotechnology and Research Institute of Agriculture and Life Science, Seoul National University, Seoul 151921, Korea

**Keywords:** Body Weight, Growth, Korean Native Chicken, Association, Thyroid Hormone Responsive Spot 14 Alpha Gene

## Abstract

**Objective:**

Thyroid hormone responsive spot 14 alpha (*THRSP*) has been used to investigate the regulation of de novo lipogenesis because the variation of *THRSP* mRNA content in the tissue affects directly the ability of that tissue to synthetize lipids. Also, this gene responds to thyroid hormone stimulation and high level of carbohydrate feeding or insulin-injection. This study was carried out to investigate variations within *THRSP* and their effects on body and carcass weights in Korean native chicken (KNC).

**Methods:**

A total of 585 chickens which represent the five lines of KNC (Black, Gray-Brown, Red-Brown, White, and Yellow-Brown) were reared and body weight data were recorded every two weeks from hatch until 20 weeks of age. Polymerase chain reaction-restriction fragment length polymorphism, DNA chips for Agilent 2100 Bioanalyzer, and Fluidigm Genotyping Technology, were applied to genotype selected markers. A linear mixed-effect model was used to access association between these single nucleotide polymorphism (SNP) markers and growth-related traits.

**Results:**

A total of 30 polymorphisms were investigated in *THRSP*. Of these, nine SNPs for loci were selected to perform association analyses. Significant associations were detected between g.-49G>T SNP with body weight at 20 weeks of age (BW20), g.451T>C SNP with growth at 10 to 12 weeks of age (GR10-12), and g.1432A>C SNP with growth at 14 to 16 weeks trait (GR14-16) and body weight at 18 weeks of age (BW18). Moreover, diplotype of the *THRSP* gene significantly affected body weight at 12 weeks of age (BW12) and GR10-12 traits. Diplotype of ht1/ht2 was favorable for BW12 and GR10-12 traits.

**Conclusion:**

These results suggest that *THRSP* can be regarded as a candidate gene for growth traits in KNC.

## INTRODUCTION

Korean native chicken (KNC) is an important genetic resource in Korea. The KNC conservation program was launched by the National Institute of Animal Science in 1994 [1,2]. After the two decades of the conservation program’s duration, the five lines of KNC, which are distinguished by plumage color, have been established and successfully released in the Korean market and are 2 to 3 times more expensive than commercial broilers [3,4]. The advantages of KNC in Korean market are that they have higher protein and lower fat contents compared to commercial broiler. However, like most indigenous chicken breeds, KNC have a relatively slower growth performance that implies a longer rearing time [1,3]. The heritability value (*h*^2^) of growth traits of KNC is high at an early age and becomes low to moderate as the chickens age [5]. Therefore, breeding strategies based on either quantitative or molecular genetics to improve growth performance of KNC are required.

Body weight in chickens can be easily observed and measured [6]. However, it is a direct indicator of growth which is a complex, and highly orchestrated phenomenon involving multiple genetic factors and environmental conditions with a host of different hormones and their receptors in various tissues [7]. Hence, it is not surprising that most of growth-related traits are regarded as complex quantitative traits.

Regulation of body weight has been an interesting topic to investigate since it is tightly related to obesity, it requires an understanding of lipid metabolism and energy balance processes [8–11]. Lipid synthesis involves multiple genes in highly complicated and integrated processes since they will maximize the storage of energy when food nutrients are abundant and repress energy expenditure when nutrients are restricted. Thyroid hormone responsive spot 14 (*THRSP*) gene, which is widely studied as a model of de novo lipogenesis regulation, encodes a small acidic *THRSP* protein. This protein acts as a transcription factor involved in control of lipogenic enzymes since it responds to thyroid hormone stimulation [12–14]. It is located in the chromosomal region affecting obesity susceptibility, and its expression is increased by carbohydrate feeding or insulin-injection [15]. In humans, *THRSP* is associated with obesity, growth and differentiation of breast cancer cells [16,17]. In chickens, *THRSP* was firstly analyzed by Cogburn et al [18] using microarray analysis that showed a differential expression of the *THRSP* sequence tag in liver of chickens genetically selected for fast or slow growth rate. It was mapped at 1q41–44 [19], where the quantitative trait locus (QTL) region for abdominal fat yield, skin fatness, and abdominal fatness were found [20–22]. The polymorphisms within this gene have been investigated using polymerase chain reaction-restriction fragment length polymorphism (PCR-RFLP) approach which revealed that they were associated with growth-related and fatness traits in chicken F_2_ intercross pedigrees [23,24]. Hence, the objective of this study was to investigate polymorphisms of *THRSP* and to evaluate their effects on the growth-related traits in KNC.

## MATERIALS AND METHODS

### Animal resources and DNA extraction

A total of 585 individuals of KNC (282 males and 303 females) comprising 68 F_1_ nuclear families ranging in size from 3 to 20 chickens were used in this study. They were divided into five lines on the basis of plumage colors (Black, 88; Gray-Brown, 110; Red-Brown, 135; White, 122; and Yellow-Brown, 130) [25]. They were reared under controlled feeding and environmental conditions in the National Institute of Animal Sciences (NIAS), Republic of Korea. This study was also performed to meet recommendations described in “The Guide for the Care and Use of Laboratory Animals” published by the Institutional Animal Care and Use Committee of the National Institute of Animal Science (2012-C-037) in Korea. Body weights were measured at every two weeks from hatch to 20 weeks of age. Descriptive statistics of body weight and growth-related traits in the KNC population can be found elsewhere [26].

For the molecular genetic analysis, blood samples were taken from wing veins using 3 mL tubes containing ethylenediaminetetraacetic acid. Moreover, these blood samples were processed to isolate genomic DNA according to the method described by Miller et al [27]. The DNA concentration was measured using NanoDrop 2000c UV-Vis Spectrophotometer (Thermo Fisher Scientific Inc., Waltham, MA, USA). Isolated DNA genome was stored in the refrigerator at −20°C to maintain DNA quality until used.

### Polymerase chain reaction and genotyping

Three pairs of primer sets and restriction enzyme information used in this study are shown in Supplementary Table S1. Two primers were designed using primer3 version 0.4.0 (http://frodo.wi.mit.edu/primer3/) based on GenBank reference NC_006088.3, and a primer pair for 9-bp indel detection that was previously reported by Wang et al [15]. The PCR was carried out in 20 μL volume containing 25 ng per μL DNA genome, 0.8 μL each primer, 1.6 μL 10 mM dNTP, 2.0 μL 10× reaction buffer, 0.2 μL Hot Start *Taq* Polymerase (GenetBio, Daejeon, Korea), and 12.6 μL double distilled water. The PCR reactions were carried out in the following steps: pre-denaturation at 94°C for 10 minutes, 35 cycles of 94°C for 30 seconds, annealing temperature (°C) for each primer for 30 seconds (Supplementary Table S1), and 72°C for 30 seconds, and followed by final extension at 72°C for 10 minutes. Reaction was performed using either GeneAmp PCR system 2700 (Applied Biosystems, Foster City, CA, USA) or C1000TM Thermal Cycler (BioRad, Hercules, CA, USA). The PCR products were visualized by 2% standard agarose gels stained with ethidium bromide (GenetBio, Korea). Initially, DNA pools containing 3 individual samples were used for PCR and sequencing to screen polymorphisms of *THRSP*. The PCR-RFLP was applied to confirm 3 single nucleotide polymorphism (SNPs) and a 3-bp indel. Approximately, 15 μL of PCR product was digested with 2 units of each restriction enzyme (Supplementary Table S1) based on the protocol provided by company (New England Biolabs Inc., Ipswich, MA, USA). Furthermore, the digested PCR product was separated on 3% agarose gels to identify genotype variations (Supplementary Figure S1). The 9-bp indel was confirmed using DNA Chip for The Agilent 2100 Bioanalyzer (Agilent Technologies Inc., Santa Clara, CA, USA) by following company protocol (Supplementary Figure S2). In addition, eleven of SNPs were genotyped using standard protocol of Fluidigm 192.24 SNPtype Genotyping Technology (Fluidigm, South San Francisco, CA, USA).

### Single marker association analysis

The phenotypic data of growth-related traits from the 585 F_1_ progeny were in normal distribution (Data not shown). Single marker association analyses between the selected DNA markers and the traits using the F_1_ progeny was conducted using the following linear mixed-model:

$${\text{Y}}_{\text{ijklmn}}=\mu +{\text{G}}_{\text{i}}+{\text{S}}_{\text{j}}+{\text{B}}_{\text{k}}+{\text{L}}_{\text{l}}+{\text{P}}_{\text{m}}+{ɛ}_{\text{ijklmn}}$$

where, Y_ijklmn_ is the phenotype of the n^th^ animal, μ is overall mean, G_i_ is the fixed effect of *THRSP* genotype i, S_j_ is the fixed effect of sex j, B_k_ is the fixed effect of batch k, L_l_ is the fixed effect of line l, and P_m_ is random additive polygenic effect of animal m, ɛ_ijklmn_ is the random residual associated with the n^th^ animal. The mean and variance for random additive polygenic effects can be defined as: P~*N*(0, **A***σ*_a_^2^), where **A** is based on relationship matrix computed from the nuclear pedigree in this study and *σ*_a_^2^ is the additive polygenic variance. The mean and variance for the residual random effect of individuals can be defined as: ɛ~*N*(0, **I***σ*_e_^2^), where **I** is the identity matrix and *σ*_e_^2^ is the residual variance.

The significance level of fixed effects in the mixed-effects model above was computed by the Wald test in ASReml program (VSN International, Hemel Hempstead, UK). Nominal p<0.05 was regarded as significant for all tests.

### Haplotype-based association analysis

**Figure 1:**
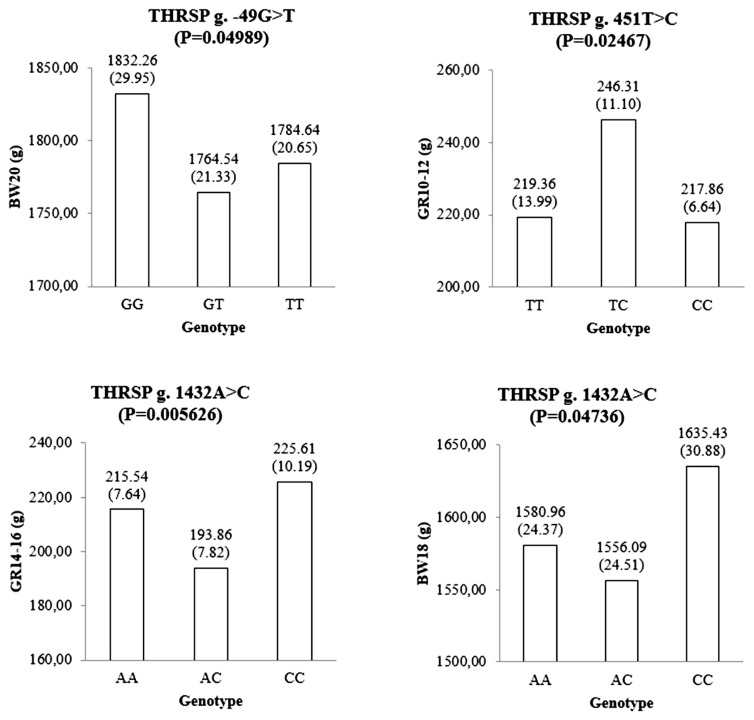
The genotypic means and standard errors of growth-related traits represent significant associations between the genotypes of the *THRSP* gene and growth-related traits. p values were calculated using a mixed-effects model analysis. BW00 is body weight at birth; BW02 is body weight at 2 weeks of age; BW04 is body weight at 4 weeks of age; BW06 is body weight at 6 weeks of age; BW08 is body weight at 8 weeks of age; BW10 is body weight at 10 weeks of age; BW12 is body weight at 12 weeks of age; BW14 is body weight at 14 weeks of age; BW16 is body weight at 16 weeks of age; BW18 is body weight at 18 weeks of age; BW20 is body weight at 20 weeks of age; GR0–2 is body weight gain at 0 to 2 weeks of age; GR2–4 is body weight gain at 2 to 4 weeks of age; GR4–6 is body weight gain at 4 to 6 weeks of age; GR6–8 is body weight gain at 6 to 8 weeks of age; GR8–10 is body weight gain at 8 to 10 weeks of age; GR10–12 is body weight gain at 10 to 12 weeks of age; GR12–14 is body weight gain at 12 to 14 weeks of age; GR14–16 is body weight gain at 14 to 16 weeks of age; GR16–18 is body weight gain at 16 to 18 weeks of age; GR18–20 is body weight gain at 18 to 20 weeks of age.

The F_1_ progeny were used to establish haplotypes at *THRSP* locus using the SNP markers showing nominal significance (p<0.05) for growth-related traits (Figure 1). The FImpute program based on pedigree information was applied to establish the haplotypes (REF. BMC Genomics 15, 478, 2014). Subsequently, the haplotype data of the F_1_ chickens were used to conduct haplotype-based association study. The effect of haplotypes on the traits of interests were evaluated by the following general linear model using the MINITAB program (MINITAB, State College, PA, USA):

$${\text{Y}}_{\text{ijklmn}}=\mu +{\text{D}}_{\text{i}}+{\text{S}}_{\text{j}}+{\text{B}}_{\text{k}}+{\text{L}}_{\text{l}}+{\text{Dam}}_{\left(\text{l},\text{m}\right)}+{ɛ}_{\text{ijklmn}}$$

Y_ijklmn_ is the phenotype of the n^th^ animal, μ is overall mean, D_i_ is the fixed effect of *THRSP* diplotype i (Table 1), S_j_ is the fixed effect of sex j, B_k_ is the fixed effect of batch k, L_l_ is the fixed effect of line l, and Dam_(l,m)_ is fixed effect of m^th^ dam within l^th^ line, ɛ is the random residual term of the model.

## RESULTS

### Detection of polymorphisms in the *THRSP* gene

Five sequences of *THRSP* were deposited in the National Center for Biotechnology Information (NCBI) website (GenBank accession numbers: KF574271-KF574275). A total of 30 polymorphisms were identified by aligning the sequences using ClustalW2 online software (http://www.ebi.ac.uk/Tools/msa/clustalw2/). These variations were spread from upstream to downstream of the gene. Eleven SNPs and an 3-bp indel at 1,355 bp from ATG were found in the flanking regions and untranslated regions of the *THRSP* gene, thirteen SNPs and an 3-bp indel at 965 bp from ATG were identified in intron, and three SNPs were also observed in the exon 1 of the *THRSP* gene. In addition, an 9-bp indel at 236 bp from start codon in the exon region of the *THRSP* gene which is identified in previous studies to be associated with body weight and abdominal fat traits in chickens was detected in present study (Table 1).

### The effects of the *THRSP* polymorphisms on growth-related traits

**Figure 2:**
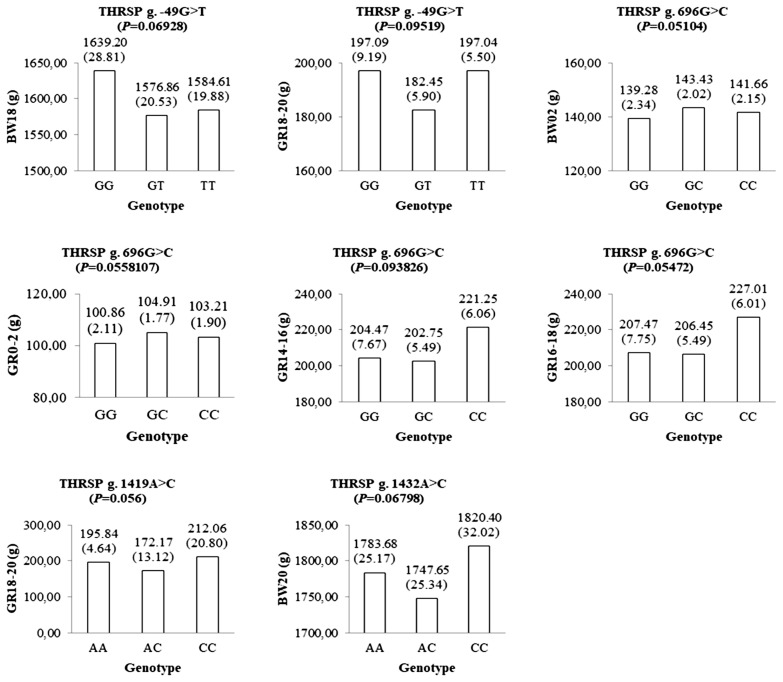
The genotypic means and standard errors of growth-related traits represent suggestive-significant associations between the genotypes of the *THRSP* gene and growth-related traits. p values were calculated using a mixed-effects model analysis. *THRSP*, thyroid hormone responsive spot 14 alpha gene.

A total of 30 polymorphisms in the *THRSP* gene have been identified in this study. Of these, nine polymorphisms were investigated to be associated with the body weight traits using a mixed-effects model in 585 F_1_ KNC progeny. Among the SNP markers listed in the Table 1, a genotype call rate less than 90% was not used for further association studies. Three SNPs showed significant association with growth-related traits (Figure 1). The g.−49G>T SNP of the *THRSP* gene was significantly associated with body weight at 20 weeks of age (BW20). Homozygote GG population had a higher BW20 than other genotypes. A SNP, namely g.451T>C, was also significantly associated with growth at 10 to 12 weeks (GR10–12). Moreover, g.1432A>C was statistically associated with growth 14 to 16 weeks and body weight at 18 weeks. The CC genotype of the g.1432A>C SNP was favorable for those traits. Additionally, four SNPs, namely g.−49G>T, g.696G>C, g.1419A>C, and g.1432A>C, were suggestively associated with some growth-related traits in F_1_ KNC population (Figure 2).

We also constructed haplotypes of the *THRSP* gene to evaluate the effect of the *THRSP* haplotype on growth-related phenotypes in the F_1_ chicken population. Haplotype and diplotype frequencies of the *THRSP* gene in the F_1_ chickens are listed in the Table 2. The results of haplotype-based association analysis indicated that there were significant association between the diplotype and BW12. Ht1/ht2 diplotype was clearly favorable for BW12 (Table 3). Additionally, significant association of the diplotype with GR10–12 was detected in the F_1_ animals. Ht1/ht2 diplotype was also highest in growth at 10 to 12 weeks of age. Ht4/ht4, where the most KNC population included in, was relatively moderate to high BW12 and GR10–12 traits (Table 3).

## DISCUSSION

In this study, nine novel SNPs and a 9-bp indel reported previously were used for the association analyses. Interestingly, the genotypic value of the heterozygotes of the markers in Figure 1 and 2 showed dominance effects (i.e., deviation from the mean value of the two homozygotes) [28]. Therefore, no SNP with significant additive effect was detected in this study.

Significant associations were detected between g.−49G>T SNP, g.451T>C, and g.1432A>C with some growth-related traits in KNC. Those SNPs were located at 5′-flanking region, intron, and 3′-flanking region of *THRSP* gene. No exonic SNP showed significant association with growth related traits. Hence, expression levels might play an important role in the phenotypic variation of chicken growth. After transcription factor binding motif analysis using MEME suite [29] and JASPAR database [30], we revealed that the position of g.−49G>T SNP is co-localized with MEF2D (myocyte enhancer factor-2D) binding motif sequence in the 5′-flanking region of the *THRSP* gene (i.e., g.−52~g.−47). Therefore, further expression studies can be worthwhile to characterize g.−49G>T SNP. Association results of the three significant SNPs and diplotypes indicated their possible role in controlling of body weight and growth traits of mature KNC.

Previous works showed that polymorphisms of the *THRSP* were associated with body weight, carcass traits, fat deposition, cholesterol and lipoprotein [15,23,24]. Particularly, A213C SNP was associated with body weight at 5 to 12 weeks of age and carcass weight, and two SNPs in 5′-flanking region were associated with body weight at hatch and BW at 28 days of age [23,24]. Indeed, the stronger effect of 9-bp indel has previously been discovered to be affecting body weight at 8 to 12 weeks of age and also carcass weight in chickens [23]. However, they were not confirmed in KNC population, except for an 9-bp indel in the exon region. There are several possible explanations for this discrepancy. First, the previously identified association may not be a direct effect of the polymorphisms but rather the result of linkage disequilibrium. Second, the effect of the polymorphisms on the body weight traits may be too small to be detected in our F_1_ nuclear families. In the case of previously reported associations found in chicken F_2_ intercross pedigrees [23,24], they only used the general linear model procedure without including an additive relationship matrix to access the association between *THRSP* and body weight traits.

In conclusion, three novel SNPs in the *THRSP* gene were associated with body weight and growth-related traits in KNC. These results provided additional support for association between polymorphisms in the *THRSP* and growth-related traits. It could be useful to be developed as candidate gene for growth traits in KNC population.

## Figures and Tables

**Table 1:** Distribution of the *THRSP* gene polymorphism in Korean native chicken

No.	SNP name	SNP ID in chicken genome1)	Location	SNP information
1.	g.−49G>T	rs14937292	5′-flanking region	Novel
2.	g.−17G>A	rs738325925	5′-flanking region	Novel
3.	g.−1T>C	rs316023211	5′-flanking region	Novel
4.	g.11A>G	rs740327674	5′-UTR	Novel
5.	g.17T>C	rs14937290	5′-UTR	Novel
6.	g.128C>T	rs735083064	Exon	Novel
7.	g.137A>G (c. 114A>G)	rs314316303	Exon	Novel
8.	g.235A>C	rs733948711	Exon	Novel
9.	9 bp indel at 236 bp from ATG	-	Exon	Wang et al [15]; Cao et al [23]; D’Andre Hirwa et al [24]
10.	g.451T>C	rs314618339	Intron	Novel
11.	g.489T>G	rs1059169337	Intron	Novel
12.	g.696G>C	rs316849944	Intron	Novel
13.	g.751C>T	rs1057579406	Intron	Novel
14.	g.774G>A	rs313687968	Intron	Novel
15.	g.785C>T	rs734655240	Intron	Novel
16.	g.800A>C	rs738084821	Intron	Novel
17.	g.814C>G	rs315982665	Intron	Novel
18.	g.818G>C	rs312478376	Intron	Novel
19.	g.868A>C	rs314685615	Intron	Novel
20.	g.899G>A	rs316757568	Intron	Novel
21.	g.911C>A	rs1059055828	Intron	Novel
22.	3 bp indel at 965 bp from ATG	rs1058294719	Intron	Novel
23.	g.1072C>T	rs1060381112	Intron	Novel
24.	g.1208C>T	rs312815759	3′-UTR	Novel
25.	g.1232A>G	rs1058578106	3′-UTR	Novel
26.	g.1357T>C	rs317692975	3′-UTR	Novel
27.	3 bp indel at 1355 bp from ATG	rs1060155017	3′-UTR	Novel
28.	g.1393G>A	rs315260792	3′-UTR	Novel
29.	g.1419A>C	rs316843064	3′-UTR	Novel
30.	g.1432A>C	rs313275625	3′-flanking region	Novel

*THRSP*, thyroid hormone responsive spot 14 alpha; SNP, single nucleotide polymorphism.

1) SNP ID in the chicken genome was collected from Ensembl website (http://asia.ensembl.org/).

**Table 2:** Haplotype and diplotype frequencies of the *THRSP* gene in the F_1_ chickens

Haplotype1)	Number	Frequency2)	Diplotype	Number	Frequency
G-C-C (ht1)	124	0.16	ht1/ht1	53	0.137
G-T-A (ht2)	95	0.122	ht1/ht2	18	0.046
T-T-C (ht3)	32	0.041	ht3/ht3	16	0.041
T-T-A (ht4)	447	0.577	ht4/ht4	196	0.505
G-T-C (ht5)	28	0.036	ht2/ht4	32	0.082
T-C-C (ht6)	50	0.064	ht5/ht6	28	0.072
			ht4/ht2	23	0.059
			ht6/ht2	22	0.057

*THRSP*, thyroid hormone responsive spot 14 alpha.

1) The 1st locus is *THRSP* g.−49G>T, the 2nd locus is *THRSP* g.451T>C, the 3rd locus is *THRSP* g.1432A>C.

2) The haplotypes with frequency less than 0.03 are dropped in this table.

**Table 3:** Effects of diplotype of the *THRSP* gene on BW12 and GR10–12 (LS means±SE)

Diplotype	BW121)	p-value	GR10–122)	p-value
ht1/ht1	877.4±39.19^ab^	0.028	142.9±27.59^a^	0.007
ht1/ht2	1,024.9±44.63^b^		278.3±31.41^c^	
ht3/ht3	1,001.1±55.19^ab^		278.3±38.85abc	
ht4/ht4	997.0±17.16^ab^		248.2±12.08^bc^	
ht2/ht4	948.9±39.46^ab^		246.0±27.78abc	
ht5/ht6	934.1±58.13^ab^		169.7±40.92abc	
ht4/ht2	1,001.9±46.17^ab^		226.3±32.50abc	
ht6/ht2	876.4±42.10^a^		155.8±29.64^ab^	

*THRSP*, thyroid hormone responsive spot 14 alpha; SE, standard error.

1) BW12, body weight at 12 weeks of age.

2) GR10–12, growth at 10 to 12 weeks of age.

a–c LS means with different superscripts within same column are significantly different at p<0.05.
