# Supplementary material for: Association of the thyroid hormone responsive spot 14 alpha gene with growth-related traits in Korean native chicken
Source: Asian-Australas J Anim Sci. 2020 Feb 25;33(11):1755–62. doi: 10.5713/ajas.19.0541 (PMC7649070; doi:10.5713/ajas.19.0541)
Supplement: Supplementary file 1 [file ajas-19-0541-suppl.pdf]

Supplementary Table 1. Primers, annealing temperature, product size and restriction enzyme details.

No.	Primer pair (5′ to 3′)	Tm (°C)	Product size (bp)	Restriction enzyme; Variation
1.	F: GCCTCCGTCACCGATCAG<br>R: CGGTCAGAACCTGCTGCAA	63	136 or 127	-; 9-bp indel
2.	F: CAGGAGGGAGCAGAGGGATAG<br>R: TTGTGTTTACCCAGCAGCAG	65	828	*Age*I; g.128C>T
3.	F: ATGCTGACCCCAAGATTGTC<br>R: TCCTTGTCATCGTGGGCTAAG	67	827	*Fok*I; 3-bp indel<br>*Alw*NI; g.868A>C<br>*Hinf*I; g.1393G>A

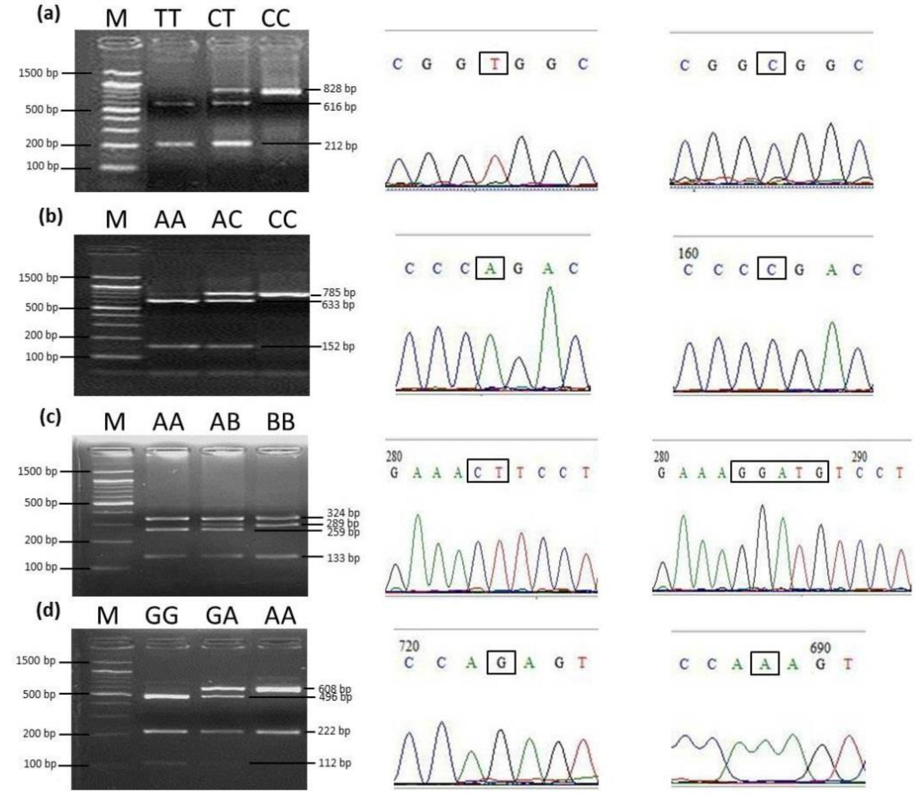

Supplementary Figure 1. The PCR-RFLP results for the variations in THRSP gene and the sequencing information. (a) The genotypes of g.128T>C SNP detected by *Age*I, (b) The genotypes of g.868A>C SNP detected by *Alw*NI, (c) The genotypes of 3 bp indel detected by *Fok*I, and (d) The genotypes of g.1393G>A SNP detected by *Hinf*I.

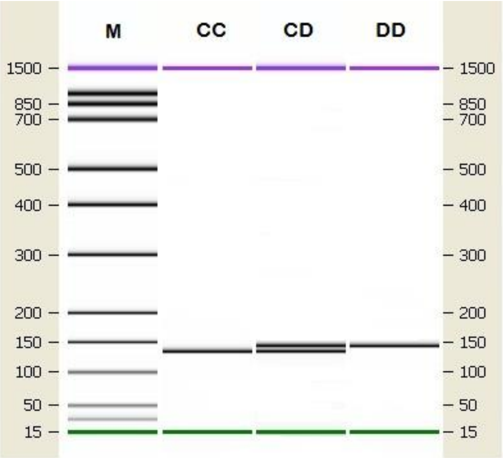

Supplementary Figure 2. The genotyping results of the 9 bp indel in exon region of the THRSP gene using Agilent DNA 1000 Technology. M: molecular marker; CC genotype (135 bp); CD genotype (144 and 135 bp); and DD genotype (144 bp).
